# Monitoring Sexually Transmitted Infections in Cervicovaginal Exfoliative Samples in Mexican Women

**DOI:** 10.3390/pathogens10121618

**Published:** 2021-12-13

**Authors:** Fabiola Hernández-Rosas, Manuel Rey-Barrera, Ulises Conejo-Saucedo, Erika Orozco-Hernández, Liliana Maza-Sánchez, Enrique Navarro-Vidal, Yasmín López-Vera, María del Carmen Ascencio-Gordillo, Mercedes Piedad de León-Bautista

**Affiliations:** 1 Biomedical Engineering Faculty, Anáhuac University, El Marques 76246, Mexico; fhernandezrosas85@gmail.com; 2 Cambrico Biotech, 41015 Sevilla, Spain; mrey@cambri.co; 3 Translational Medicine, Vanguard and Technology Transfer Sector, Human Health Department, Central ADN Laboratories, Morelia 58341, Mexico; conejet@me.com (U.C.-S.); eorozco@centraladn.com (E.O.-H.); lilianamaza@yahoo.com.mx (L.M.-S.); enrique.navarro@centraladn.com (E.N.-V.); 4 Hospital de la Mujer de la Secretaría de Salud de Michoacán, Morelia 58295, Mexico; lopezvyas@yahoo.com.mx (Y.L.-V.); m.ag62@hotmail.com (M.d.C.A.-G.); 5 Escuela de Medicina, Universidad Vasco de Quiroga, Morelia 58090, Mexico

**Keywords:** sexually transmitted infection, PCR multiplex detection, sustainable development goals

## Abstract

Background. Globally, Sexually Transmitted Infections (STIs) are a major cause of morbidity in sexually active individuals, having complications in reproduction health and quality of life. In concordance with the Sustainable Development Goals (SDG), the study aimed to investigate the prevalence of *Candida* spp., *Ureaplasma* spp., *Trichomonas vaginalis*, *Neisseria gonorrhoeae*, *Chlamydia trachomatis*, HSV, and *Mycoplasma* spp. from cervicovaginal samples and to correlate them with the gynecological history of the patients. Methods. Our analytical, prospective, and cross-sectional study included 377 women who participated in a reproductive health campaign during 2015–2016. Anthropometric and gynecological variables were obtained. Cervicovaginal specimens were collected and analyzed with a multiplex in-house PCR to detect *Candida* spp., *Ureaplasma* spp., *Trichomonas vaginalis*, *Neisseria gonorrhoeae*, HSV, *Mycoplasma* spp., and *Chlamydia trachomatis*. Results. The positive cases were 175/377 (46.4%) to at least one of the microorganisms. The most frequent pathogen detected in this population was *Ureaplasma* spp. (*n* = 111, 29.4%), followed by *Mycoplasma* spp. (*n* = 56, 14.9%) and *Candida* spp. (*n* = 47, 12.5%); 33.7% of the positive cases were single infections, whereas 12.7% had coinfection. The multiplex PCR assay was designed targeting nucleotide sequences. Conclusions. Our data demonstrated that monitoring STIs among asymptomatic patients will encourage target programs to be more precisely and effectively implemented, as well as make these programs more affordable, to benefit society by decreasing the prevalence of STIs.

## 1. Introduction

Sexually Transmitted Infections (STIs) and Bacterial Vaginosis (BV) represent a concerning important health problem throughout the world. Globally, the most common STIs are caused by *Chlamydia trachomatis* (CT), *Neisseria gonorrhoeae* (NG), and *Trichomonas vaginalis* (TV) [1,2]. In some cases, these infections are asymptomatic, masking the pathogen leading to fertility complications and consequences in sexual and maternal–fetal health. Moreover, the proinflammatory microenvironment caused by these pathogens increases the risk of acquiring other infections such as HIV [3].

The STI epidemic is dynamic; thus, for gathering robust data, these diseases and infections need to be monitored to allow target programs to be more precisely and effectively implemented to benefit society. On this concerning fact, to improve the sexual health of men and women, the Sustainable Development Goals (SDG) were postulated with the clear objective to radically reduce the prevalence of STIs and deaths caused by these infections [4]. As part of these strategies, we are encouraged to improve the screening methods and to make them more widely available to the population, as current molecular assays only offer solutions with high costs of the commercial kits [5], the usage of sophisticated platforms [6,7] and expensive shipping costs. Therefore, based on its global targets, the ultimate objective pursued with this project is the development of a diagnostic kit for STI and BV that will allow establishing the specific causal infectious agents with a single assay. Nevertheless, to achieve this goal, one of the particular objectives was to describe the prevalence of STI and BV in asymptomatic women. Therefore, in this study, we showed the correlation of gynecological history with the prevalence of *Candida* spp., *Ureaplasma* spp., *Trichomonas vaginalis*, *Neisseria gonorrhoeae*, *Chlamydia trachomatis*, HSV, and *Mycoplasma* spp. from cervicovaginal samples, though a multiplex in-house PCR which has a potential clinical utility.

## 2. Materials and Methods

### 2.1. Study Subjects and Selection Criteria

An analytical, prospective, and cross-sectional study was conducted with 377 women from the state of Michoacán, located in central Mexico. They accepted to participate in the screening campaign to detect *Candida* spp., *Ureaplasma* spp., *Trichomonas vaginalis*, *Neisseria gonorrhoeae*, *Chlamydia trachomatis*, HSV, and *Mycoplasma* spp. during the period from December 2014 to June 2015. All the women were aged 15 to 65 years old and were apparently healthy and asymptomatic at the time of sample collection. The anthropometric information and gynecological history were obtained from a standardized individual questionnaire.

### 2.2. Ethical Statement

The study was approved by the medical and ethical research committee at Hospital de la Mujer de la Secretaría de Salud de Michoacán, with project number 090. The protocols were carried out according to the Declaration of Helsinki and the current health laws in Mexico. The informed consent was signed by all the participants before performing any clinical procedure.

### 2.3. Specimen Collection

All clinical cervicovaginal exfoliative samples were obtained by scraping the epithelium with a nylon cytobrush (Puritan Medical Products, Guilford, ME, USA). Every specimen was stored in Universal Transport Medium (Copan Diagnostics, Murrieta, CA, USA) at room temperature and processed in the molecular diagnostic laboratory Central ADN in Morelia, Michoacán, Mexico.

### 2.4. DNA Extraction

The extraction of the total DNA was carried out by using Instagene Matrix (Bio-Rad, Hercules, CA, USA), following the manufacturer’s instructions. Briefly, 200 µL of the sample was transferred to 200 µL tubes. Then, the samples were centrifuged at 14,000 rpm for 1 min. The pellet was washed twice with PBS-Tween 20. Finally, 50 µL of Instagene Matrix was added to the pellet and incubated at 56 °C for 30 min. The sample was boiled to 100 °C for 8 min. The supernatants were stored at −20 °C until their use.

### 2.5. Primer Design

The DNA sequences of *Candida* spp., *Ureaplasma* spp., *Trichomonas vaginalis*, *Neisseria gonorrhoeae*, HSV, and *Mycoplasma* spp. were obtained from GenBank (accession numbers listed in Table 1). Every gene-specific primer pair was designed as a multiplex PCR assay with non-dimerizing primer set combinations by using the MPprimer software [8]. The primer sequences for the PCR assay of bacterial, parasites, and viral pathogens are described in Supplementary Table S1. For the detection of *Chlamydia trachomatis,* we used a pair of primers previously reported [9].

### 2.6. Positive Control Strains, Sequence Analysis, and Standardization

As positive controls, well-characterized clinical samples of the cervix of *Candida* spp., *Ureaplasma* spp., *Trichomonas vaginalis*, *Neisseria gonorrhoeae*, *Chlamydia trachomatis*, HSV, and *Mycoplasma* spp. were kindly shared by Hospital de la Mujer de la Secretaría de Salud de Michoacán. Single PCR was performed using well-characterized clinical samples to assure specificity. Thus, to confirm the results, the amplified products were purified and sequenced with forward and reverse primers at Macrogen (Seoul, Korea). The sequences were blasted against available fragments in the gene bank to identify the microorganism.

### 2.7. Positive Control Constructs

Once we confirmed the specificity of the primers, the specific DNA fragments were cloned into pGEM vector to generate the constructs pGEM/CA, pGEM/UP, pGEM/TV, pGEM/NG, pGEM/CT, pGEM/HSV, and pGEM/MY as positive synthetic controls. The plasmids were introduced into *E. coli DH-5α* and purified using alkaline lysis [10]. The inserts were verified by a PCR reaction and confirmed by automated sequencing analysis at Macrogen (Seoul, Korea), using the forward and reverse probes to the corresponding pathogen. The sequences were blasted in the gene bank to identify the microorganism.

### 2.8. PCR Assays Conditions

The single PCR reactions were carried out using QIAGEN^®^ Multiplex PCR Kit (206145, Qiagen, Hilden, Germany), in a final volume of 10 µL prepared with PCR master mix buffer [1×]f, and 2 µL, (~100 ng/µL) of total nucleic acid extraction. Due to the variability of the Tm of primers, and to increase the specificity, we performed a touch-down PCR with a Hot Start activation step at 95 °C for 5 min; followed by 35-cycle steps, consisting of denaturation step at 95 °C for 1 min, after, the first 16 cycles at 65 °C for 1 min; for the next 19 cycles, the annealing temperature decreased 0.3 °C each. The extension step was at 72 °C for 45 s. The PCR steps were carried out using a standard Veriti™ Thermal Cycler (LS4375786, Thermo Fisher Scientific, Waltham, MA, USA).

To resolute the PCR, the total reaction mixture was subjected to electrophoresis on a 2.5% agarose gel dyed with Red Safe (21141, iNtRON, Seongnam, Korea). The bands were visualized on a UV transilluminator and photographed (ENDURO™ GDS, LabNet international, Edison, NJ, USA).

Once the single-target PCR system was successful, we performed an in-house multiplex PCR assay with the confirmed probe sets to detect *Candida* spp., *Ureaplasma* spp., *Trichomonas vaginalis*, *Neisseria gonorrhoeae*, *Chlamydia trachomatis*, HSV, and *Mycoplasma* spp. Human Actin primers were used as an internal control, 5′ACCGAGCGCGGCTACAG3′ and 5′CTTAATGTCACGCACGATTTCC3′ (ShineGene Molecular Biotech) forward and reverse, respectively.

To reinforce the results with the multiplex assay, the detected amplicons were purified to perform an automated sequencing analysis at Macrogen (Seoul, Korea), using the specific probes to the corresponding pathogen, forward and reverse, respectively. The sequences were blasted in the gene bank to identify the microorganism.

### 2.9. Statistical Analysis

Statistical analyses were performed using SPSS version 25.0 (IBM Statistics, Armonk, NY, USA). Descriptive analyses of categorical variables were expressed as frequency and percentages such as age group, gynecological history, and STI results. The quantitative continuous variables were expressed as means ± standard deviation. Pearson chi-square test (Chi^2^) was used to assess relationships between variables and the STI using a significance level of *p* < 0.05 (two-tailed). The conditional logistic regression was used to estimate odds ratios (OR) of factors associated with positive multiplex PCR results with 95% confidence intervals (CI). The multivariate logistic regression included the effects of age and clinical variables.

## 3. Results

### 3.1. Multiplex PCR Assay

**Figure 1:**
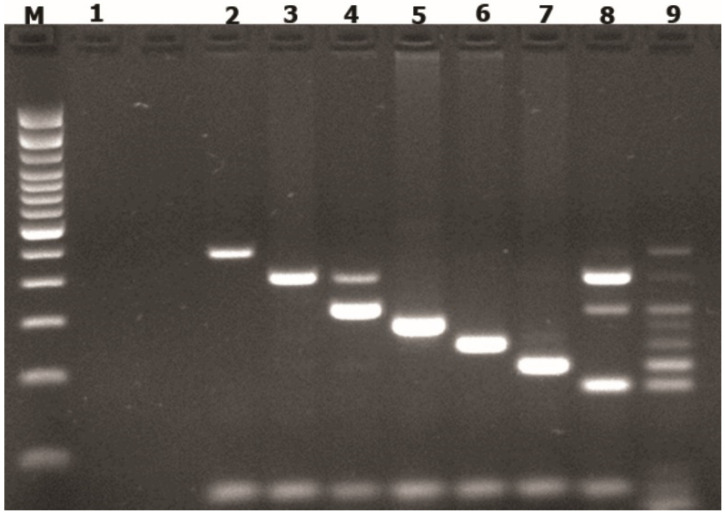
Multiplex End-point PCR. Gel electrophoresis (2.5% agarose) of PCR products from well-characterized samples to detect *Candida* spp., *Ureaplasma* spp., *Trichomonas vaginalis*, *Neisseria gonorrhoeae*, *Chlamydia trachomatis*, HSV and *Mycoplasma* spp. M, Ladder 100 bp marker (Invitrogen, Waltham, MA, USA); (1) negative control (molecular grade water); (2–8) well-characterized samples: (2) *Candida* spp. (473 bp), (3) *Ureaplasma* spp. (397 bp) and *Trichomonas vaginalis* (308 bp); (4) *Trichomonas vaginalis* (308 bp); (5) *Neisseria gonorrhoeae* (278 bp); (6) *Chlamydia trachomatis* (241 bp); (7) HSV (205 bp); (8) *Mycoplasma* spp. (184 bp), *Ureaplasma* spp. (397 bp) and *Trichomonas vaginalis* (308 bp); (9) pool of cloning plasmids as a positive control. The band of ~65 bp corresponds to actin beta as an internal control.

Based on the target species, the designed primers were specific to detect Candida spp., Ureaplasma spp., Trichomonas vaginalis, Neisseria gonorrhoeae, HSV, *Mycoplasma* spp. and *Chlamydia trachomatis*. The multiplex PCR assay was performed using a touch-down PCR assay (Figure 1). All the well-characterized clinical samples were correctly identified, and to demonstrate the standardization, we performed the experiments in triplicates, indicating a high degree of reproducibility of this multiplex assay. It is relevant that in some cases, the well-characterized clinical samples presented an unidentified amplicon, due to the donated clinical samples being tested with single-target PCR to detect just one pathogen. Thus, to solve this incognito, unknown bands, 397 bp (Figure 1, line 4, 8) and 308 bp amplicon (Figure 1, line 8) were automated sequenced with forward and reverse primers at Macrogen (Seoul, Korea). The reattributed sequences were blasted [11] against available fragments in the gene bank to identify the microorganism. The results matched with *Ureaplasma* and *Trichomonas vaginalis,* both pathogens included in our test. These sequencing results showed that our method identified more than one pathogen in the same sample. These results demonstrate the standardization parameters of our assay. Further experiments must be performed to complete the validity of the test.

### 3.2. Clinical Findings

A total of 377 cervical samples from women aged 15 to 65 years old were analyzed. The gynecological history of the patients is detailed in Table 2. The average age of the population of the study was 37.7 ± 11.8 years. Most of the patients were in the age group 15 to 20 years (64.2%) and 21 to 25 years (20.2%). Of all patients, 34.5% had multiple sexual partners and 6.1% had their first sexual intercourse before 15 years old. A history of previous STI was found in 11.4% of the patients. The gynecological examination revealed that most of the patients had a normal cervix (41.7%), and only 11.7% had a cervical lesion (Table 2). The most frequent abnormality was an abnormal vaginal discharge (15.9%). Of all of the patients, 50.9% had at least one contraceptive method. Smoking habit was described in 15.9% of the study population. Only 15 patients underwent cervical cytology, the results are shown in Table 2.

### 3.3. Detection of STI in Cervical Samples Using Multiplex PCR Assay

**Figure 2:**
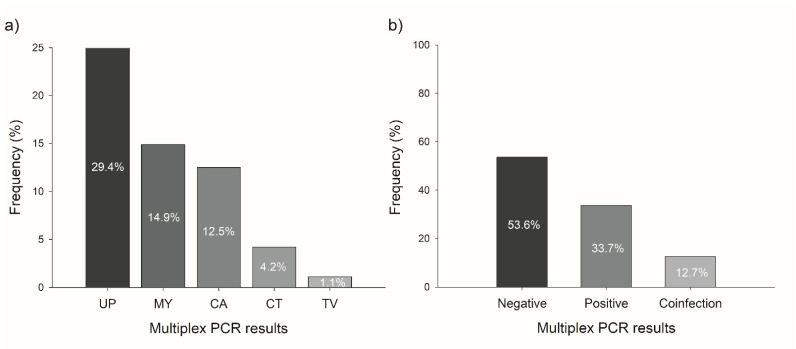
Results of multiplex PCR by pathogens. (**a**) Pathogens detected by multiplex PCR. (**b**) Frequency of positive and coinfection results. Abbreviations: UP, *Ureaplasma* spp.; MY, *Mycoplasma* spp.; CA, *Candida* spp.; CT, *Chlamydia trachomatis;* TV, *Trichomonas vaginalis*.

The multiplex PCR panel allowed the identification of *Candida* spp. (473 bp), *Ureaplasma* spp. (397 bp), *Trichomonas vaginalis* (310 bp), *Neisseria gonorrhoeae* (278 bp), *Chlamydia trachomatis* (241 bp), HSV (205 bp), and *Mycoplasma* spp. (184 bp) (Figure 1). As part of the quality control and assurance of our multiplex test and having internal consistency, a random selection of positive samples to different pathogens was automated sequenced, demonstrating a 100% of concordance with the genes detected in GeneBank. The positive cases were 175/377 (46.4%) to at least one of the microorganisms included in the test. The most frequent pathogen detected in this population was *Ureaplasma* spp. (*n* = 111, 29.4%), followed by *Mycoplasma* spp. (*n* = 56, 14.9%), *Candida* spp. (*n* = 47, 12.5%), *Chlamydia trachomatis* (4.2%), and *Trichomonas vaginalis* (1.1%) (Figure 2a). We did not find positive samples for *N. gonorrhoeae* or HSV. Of the positive cases, 33.7% were single infections, whereas 12.7% had coinfection with other pathogens, mostly: *Candida albicans/Ureaplasma* spp. (4.5%), *Ureaplasma* spp./*Mycoplasma* spp. (3.4%), and *Ureaplasma* spp./*Chlamydia trachomatis* (2.1%). Of all samples, 53.6% were negative (Figure 2b).

**Figure 3:**
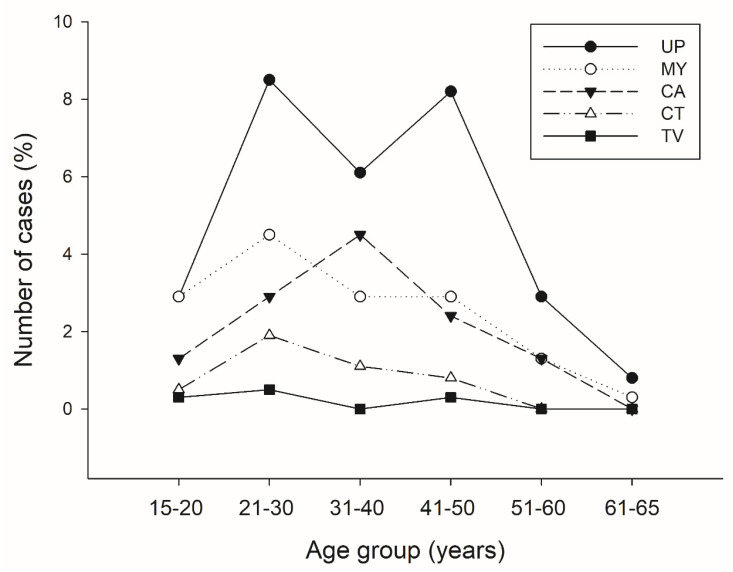
Pathogens detected by multiplex PCR stratified by age 15–20, 21–30, 31–40, 41–50, 51–60, 61–65 years. Abbreviations: UP, *Ureaplasma* spp.; MY, *Mycoplasma* spp.; CA, *Candida* spp.; CT, *Chlamydia trachomatis;* TV, *Trichomonas vaginalis*.

The samples with positive results were stratified according to age group (Figure 3). We found the highest frequency of *Ureaplasma* spp., *Mycoplasma* spp., and *Trichomonas vaginalis* at the 21 to 30 and 41 to 50 years age group. Nevertheless, in the 31 to 40 years age group the frequency of *Candida* spp. was higher (Figure 3). *Chlamydia trachomatis* frequency was common at the age of 21 to 30 years (Figure 3).

**Figure 4:**
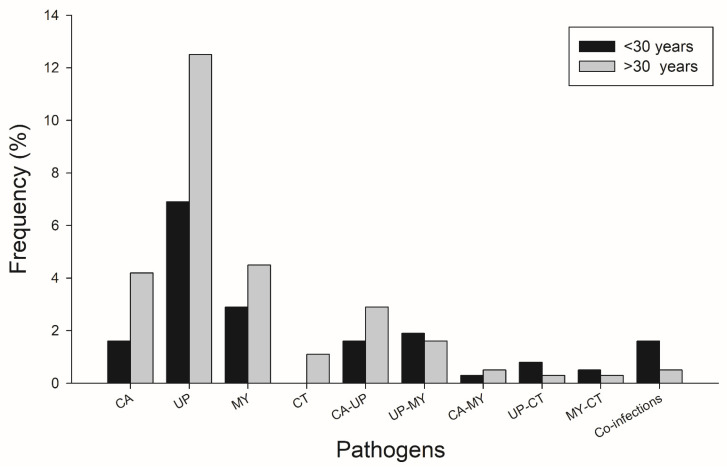
Age-Stratified Multiplex PCR Results. Abbreviations: UP, *Ureaplasma* spp.; MY, *Mycoplasma* spp.; CA, *Candida* spp.; CT, *Chlamydia trachomatis;* TV, *Trichomonas vaginalis*.

We compared two different age groups, less than 30 years and above 30 years, with the multiplex PCR results (Figure 4). The most frequent microorganisms detected were *Candida* spp., *Ureaplasma* spp., *Mycoplasma* spp., and *Chlamydia trachomatis* in women older than 30 years. In the case of coinfection, the positive results were more frequent in women younger than 30 years of age (Figure 4).

Most of the patients with a positive result were asymptomatic. However, during the pelvic examination, the most significant signs and symptoms were cervicitis, abnormal vaginal discharge, and inflammatory changes in the cervix (Table 3). The last was the most frequent macroscopic found in samples positive to *Ureaplasma* spp. and *Mycoplasma* spp.; 5.8% and 3.8%, respectively, followed by an increased vaginal discharge, with 1.6% and 2.1%, respectively (Table 3). Comparing symptomatic or asymptomatic patients concerning the presence of any pathogen, we did not find significant differences between the two groups (*p* > 0.05) (Table 3). Due to the absence of *N. gonorrhoeae* and HSV positive samples, we could not correlate this information with the pelvic examination.

Furthermore, we analyzed the potential risk factors associated with the presence of at least one of the microorganisms tested in the multiplex panel. A univariate logistic regression model for sexual behavior and an adjusted multivariate model for age and clinical variables were explored. We found that having multiple sexual partners represents a risk factor for a positive diagnostic test in the study population (OR = 1.205; 95% CI = 1.016–1.454; *p* = 0.043) (Table 4). According to the Pap smear results and the multiplex PCR test, two patients with LSIL + HPV and one patient who had LSIL + HPV + CIN1 were positive to *Mycoplasma* spp. (data not shown).

Ultimately, we independently explored the risk factors associated with positive results considering the most frequent pathogens found in our study. Our results demonstrated that the age of first sexual intercourse before 15 years represents a risk factor for acquiring an infection by *Ureaplasma* spp. (OR = 3.184; 95% CI = 1.338–7.574; *p* = 0.009), meanwhile, above 15 years is a protective factor for *Chlamydia trachomatis* infection (OR = 0.881; 95% CI = 0.825–0.042). Moreover, we showed that multiple sexual partners represent a risk factor for *Mycoplasma* spp. infection. (OR = 1.281; 95% CI = 1.050–1.321; *p* = 0.042) (Table 5).

## 4. Discussion

The Sustainable Development Goals (SDG) were postulated to reduce STIs and deaths caused by these infections, to improve the sexual health of men and women [4]. Therefore, our social commitment is motivated by the socialization of technologies, to make them achievable for all the social sectors. In this sense, STIs are highly prevalent pathologies in our country and other regions, particularly affecting poor women strata. This social context presents severe clinical outcomes since a high percentage are asymptomatic [12], there is a lack of information [13,14] to differentiate pathological from physiological situations, and there is an absence of medical check-ups and affordable screening strategies. All these causes can result in complications such as pelvic inflammatory disease (PID), which can cause infertility or ectopic pregnancy, complications derived from the *Chlamydia trachomatis* and *Neisseria gonorrhoeae* infection; nevertheless, more than 70% of the cases have an unknown etiology. Many studies have found different pathogens that cause non-gonococcal or non-chlamydial symptoms. Other pathogens involved are Mollicutes such as *Ureaplasma urealyticum* and *Ureaplasma parvum.* All of these are correlated with PID and infertility, as well as urethritis in men [15].

The WHO Global Strategy of the Health Sector on STIs 2016–2021 estimated 357 million new cases per year worldwide among people aged 15 to 49 years. The prevalence of some viral STIs is equally high, with an estimated 417 million people infected [16]. The prevalence of these STIs varies by region and gender. These epidemics have a profound impact on the health and lives of children, adolescents, and adults around the world, causing fetal and neonatal deaths, infertility, and higher risk of HIV, among other health problems.

Our study showed an overall rate of STI-positive cases (46.4%), higher in women aged 41–50 years, a frequent percentage described in a diversity of populations [17,18,19]. In this sense, Kim et al. demonstrated rates of STI-positive status of 49.2% and showed a peak distribution in those 40–49 years old, which highlights comparable results with our study population [19].

Here, we identify the most prevalent pathogens: *Ureaplasma* spp., Mycoplasma spp., Candida spp., and Chlamydia trachomatis. The prevalence of *Ureaplasma* spp. and *Mycoplasma* spp. in sexually active female populations from various regions ranged from 12 to 64% and 1 to 16%, respectively [20]. In a similar study realized by Kim et al., *Ureaplasma* spp. was the most common STI in Korean women, with an overall prevalence of 32.5%, of which 50% were under the age of 30 [10]. Compared with our study, the prevalence of *Ureaplasma* spp. was minor (29.4%); in contrast, we found that most of the positive cases of *Ureaplasma* spp. appeared in women older than 30 years. The reports concerning the prevalence of these pathogens are heterogeneous among populations.

On the other hand, the prevalence of *Chlamydia trachomatis* in our study was 4.2%, similar to a recent epidemiological Mexican study realized with 2352 women of Central Mexico, which reported an estimated prevalence of 4.3% of this pathogen [21]. It is well described that *Chlamydia trachomatis* is one of the most relevant pathogens impacting global health. Here, the data demonstrated a similar prevalence shown in Latin America [22]. This fact reveals the importance of epidemiological surveillance at the regional level. According to SDG, the prevalence and incidence must decrease in the coming years; therefore, affordable screening tests should be implemented or improved. Moreover, the highest STI prevalence occurs mainly between 15 to 24 years old, and we reiterated this information. During late adolescence age, it is common to have frequent sexual [23] contact with many sexual partners, increasing the risk of acquiring any STI. Here we found that having sexual intercourse after 15 years old is a protective factor to avoid *Chlamydia trachomatis* infection; on the contrary, this factor becomes dangerous when the individual has precocious sexual onset. Indeed, STI screening methods are one of the most important efforts to improve by 2030 [4], but also educational campaigns to strengthen the global hits in SDG should be applied.

Regarding *Trichomonas vaginalis*, the prevalence in our study was 1.1%. *Trichomoniasis* is a notable disease in Mexico; however, epidemiological data reported by the Health Societies do not represent the real magnitude of these STIs because there is no total coverage of public health services and because many women are asymptomatic [24]. In addition, there is scarce information available in Mexico about the prevalence of *Trichomonas vaginalis*, which is limited to local studies conducted in a few cities in Mexico. In this respect, a Mexican report encouraged to determine the frequency of nine sexually transmitted pathogens, coinfections, and risk factors in patients attending obstetrics and gynecology clinics in Jalisco, in western Mexico. The results showed a higher prevalence of 14.2% compared with our results [25].

While the prevalence of *T. vaginalis* in Latin America is around 11–15% [26], the CDC estimated a minor prevalence of *T. vaginalis* of 1.6% in USA women [27]. These data show a marked variability and regionalization of *T. vaginalis* transmission among countries.

Coinfections were not the most prevalent cases, with 12.7%, with similar results (12.8%) in Cervical Cytology of Samples using homolog PCR techniques [17]. Another common finding was the coinfections: *Candida* spp. with *Ureaplasma* spp. and *Mycoplasma* spp., followed by *Chlamydia trachomatis* with *Ureaplasma* spp. and *Mycoplasma* spp. in patients younger than 30 years old. Similar results were reported in patients with urogenital infections [28]. Even though the microorganisms belonging to the genus *Ureaplasma* and *Mycoplasma* are not part of the normal urogenital microbiota, they spread through sexual contact and are associated with chronic non-gonococcal urethritis (NGU) and preterm delivery. Hence, detecting asymptomatic patients is mandatory because of the sexual health and maternal–fetal implications [29]. There is controversy because the asymptomatic carriage of these bacteria is regular, and the majority of individuals will not develop signs or symptoms. Thus, this type of screening test for women should be analyzed to avoid antimicrobial resistance. Furthermore, we should continue performing extensive analyses to solve issues regarding bacterial load and symptoms [30].

*Candida* spp. is a microorganism that is part of the commensal microbiota, and it is related to vulvovaginal candidiasis (CVV), a clinical condition characterized by an infection of the genital, vulvar and vaginal mucosa. The disease is a consequence of factors that allow the excessive proliferation of yeast [31]. Although this pathology is not classified as an STI, it is a condition that favors the acquisition of another infection. In our study, this microorganism was the third most prevalent pathogen, and associated with coinfection with *Chlamydia trachomatis* and *Mycoplasma* and *Ureaplasma*. This coinfection was described previously [17,28]. Due to that *Candida* spp. was detected in coinfection with these pathogens, the main key questions are formulated. This yeast inhibits growths or the virulence of other pathogens, perhaps providing chemical resources for bacterial survival in the microenvironment, or promoting microorganism dissemination [32]. Our report has some limitations since we designed the panel test with no specific detection of *Candida* species. Thus, a supplementary test needs to be carried out to differentiate species of *Candida*.

Our study was focused on clinically healthy women with asymptomatic infection, a risk group with consequences in reproductive and maternal–fetal health, but also a higher risk of acquiring other types of infections such as HIV (human immunodeficiency virus) [33,34,35]. The use of NAATs is considered the gold standard tests to detect *Chlamydia trachomatis* and *Neisseria gonorrhoeae*; they are recommended by the CDC because of their high specificity and sensitivity when we compared them to traditional techniques [29,36]. Hence, our panel described the most prevalent STIs such as *Chlamydia trachomatis* (CT), *Candida* spp., Ureaplasma spp., Mycoplasma spp., and *Trichomonas vaginalis* (TV).

In summary, it is necessary not only to encourage the capacity of laboratories but also to design and introduce affordable STI diagnostics and sexual educational campaigns, for different uses such as detecting high-risk groups, monitoring treatment, surveillance studies, quality assurance of laboratory tests, and research. Finally, we emphasized that monitoring STIs among asymptomatic patients will encourage the implementation of target programs more precisely and effectively, as well as make these programs more affordable to benefit society and decrease the prevalence of STIs.

## Figures and Tables

**Table 1:** GenBank accession numbers to design the multiplex PCR assay.

Pathogen	Gene	Amplicon Size	Gene Bank Access	Reference
*Mycoplasma* spp.	16S rRNA	184	AY466443.1 MN149400.1	In-house
*Ureaplasma* spp.	16S rRNA	397	EU932691.1	In-house
*Candida* spp.	18S rRNA	473	X53497.1	
*Chlamydia trachomatis*	CP	241	CP054431.1	[9]
*Neisseria gonhorroeae*	pJD1 (cppb)	278	AP018387.1	In-house
*Trichomonas vaginalis*	18S rRNA	310	KX061409.1	In-house
Human Herpes Virus	DNA POL	205	MH697529.1 MH697449.1	In-house

16S rRNA, 16S ribosomal RNA; 18S rRNA, 18S ribosomal RNA CP; *Chlamydia trachomatis* plasmid; pJD1 (cppb); Cryptic plasmid protein B; DNA POL, DNA polymerase.

**Table 2:** Gynecological history (*n* = 377).

Sexual History	*n*	%
Multiple sexual partners	130	34.5
AFSI < 15 years	23	6.1
Age group		
15–20 years	242	64.2
20–25 years	76	20.2
25–30 years	30	8.0
>30 years	6	1.6
Infection		
History of STI	43	11.4
Gynecologic-obstetric history		
Puerperio	0	0.0
Postmenopause	36	9.5
Contraception		
Oral contraception	17	4.5
Injectable contraceptives	8	2.1
Subdermal Implant	2	0.5
IUD	12	3.2
Hysterectomy	18	4.8
BTL/BPS	135	35.8
Gynecological examination		
Normal cervix	158	41.9
Abnormal cervix	3	0.8
Cervix lesion	43	11.4
Cervicitis	12	3.2
Increase in vaginal discharge	60	15.9
Intermenstrual bleeding	2	0.5
Cervical and vaginal discharge	8	2.1
Cervicitis and vaginal discharge	23	6.1
Inflammatory changes	68	18
Health maintenance		
Tobacco	60	15.9
Cervical cytology		
Without PAP	362	96.0
LSIL + HPV	6	1.6
LSIL + HPV+ CIN1	8	2.1
LSIL + CIN2	1	0.3

AFSI, Age at First Sexual Intercourse; STI, Sexually Transmitted Infections; BTL/BPS, bilateral tubal ligation, or bilateral partial salpingectomy; IUD, intrauterine device; PAP, Papanicolaou test; HPV, Human Papillomavirus; LSIL, low-grade squamous intraepithelial lesion; CIN, Cervical Intraepithelial Neoplasia.

**Table 3:** Frequency of *Ureaplasma* spp., *Mycoplasma* spp., *Candida* spp., and *Chlamydia trachomatis* in symptomatic and asymptomatic patients.

	*Ureaplasma* spp.			*Mycoplasma* spp.			*Candida* spp.			*Chlamydia trachomatis*		
Signs and Symptoms	− *n* (%)	+ *n* (%)	*p* value	− *n* (%)	+ *n* (%)	*p* value	− *n* (%)	+ *n* (%)	*p* value	− *n* (%)	+ *n* (%)	*p* value
Asymptomatic	182 (48.3)	79 (21.0)	0.351	228 (60.5)	33 (8.8)	0.193	229 (60.7)	32 (8.5)	0.552	249 (66.0)	12 (3.2)	0.789
Cervicitis	8 (2.1)	4 (1.1)	0.125	11 (2.9)	1 (0.3)	0.172	11 (2.9)	1 (0.3)	0.412	12 (3.2)	0 (0.0)	0.305
Vaginal discharge	30 (8.0)	6 (1.6)	0.087	28 (7.4)	8 (2.1)	0.120	29 (7.7)	7 (1.9)	0.286	34 (9.0)	2 (0.5)	0.176
Inflammatory changes	46 (12.2)	22 (5.8)	0.064	54 (14.3)	14 (3.7)	0.071	61 (16.2)	7 (1.9)	0.078	66 (17.5)	2 (0.5)	0.081

Chi^2^ test was used to compare between groups. A *p*-value < 0.5 was considered significant.

**Table 4:** Risk factors for positive PCR results (*n* = 377).

Variable	PCR Negative	PCR Positive	OR (95% CI)	*p*-value	OR (95% CI) ^±^	*p*-Value ^±^
AFSI < 15 years						
No	194 (51.5)	160 (42.4)	Ref.		-	
Yes	8 (2.1)	15 (4.0)	2.273 (0.940–5.499)	0.062	2.055 (0.839–5.037)	0.115
History of STI						
No	176 (46.7)	158 (41.9)	Ref.			
Yes	26 (6.9)	17 (4.5)	1.373 (0.718–2.625)	0.336	1.303 (0.683–2.488)	0.422
MSP						
No	142 (37.7)	105 (27.9)	Ref.			
Yes	60 (15.9)	70 (18.6)	1.267 (1.021–1.571)	**0.036**	**1.205 (1.016–1.454)**	**0.043**
Tobacco habit						
No	173 (45.9)	144 (38.2)	Ref.			
Yes	29 (7.7)	31 (8.2)	0.779 (0.448–1.353)	0.374	0.848 (0.483–1.490)	0.567

AFSI, age of first sexual intercourse; MSP, multiple sexual partners; STI, Sexually Transmitted Infections; OR, odds ratios; CI, confidence intervals. Chi^2^ test was used to compare between groups. A *p*-value < 0.5 was considered significant. ± ORs and *p*-values adjusted for age and clinical variables. Ref; Reference of the logistic regression models formed to identify independent risk factors associated with positive PCR results.

**Table 5:** Analysis of risk factors associated to multiplex PCR results (*n* = 377).

Pathogen	Yes *n* (%)	No *n* (%)	OR (CI 95%)	*p*-Value	OR (CI 95%) ^±^	*p*-Value ^±^
ASFI < 15 years						
UP	13 (3.4)	98 (26.0)	**3.396 (1.442–7.998)**	**0.003**	**3.184 (1.338–7.574)**	**0.009**
MY	5 (1.3)	51 (13.5)	1.650 (0.587–4.642)	0.338	1.343 (0.455–3.963)	0.593
CA	3 (0.8)	44 (11.7)	1.057 (0.302–3.703)	0.931	1.049 (0.306–3.594)	0.939
CT	**0 (0.0)**	16 (4.2)	**0.936 (0.911–0.962)**	0.297	**0.881 (0.825–0.042)**	0.337
History of STI						
UP	12 (3.2)	99 (26.3)	1.088 (0.537–2.206)	0.814	1.048 (0.517–2.126)	0.897
MY	5 (1.3)	51 (13.5)	1.370 (0.515–3.645)	0.527	1.243 (0.460–3.362)	0.668
CA	3 (0.8)	44 (11.7)	2.023 (0.600–6.820)	0.247	2.037 (0.599–6.924)	0.254
CT	3 (0.8)	13 (3.4)	0.540 (0.148–1.977)	0.345	0.465 (0.124–1.743)	0.256
Multiple sexual partners						
UP	43 (11.4)	68 (18.0)	0.769 (0.485–1.217)	0.261	0.811 (0.506–1.300)	0.385
MY	27 (7.2)	29 (7.7)	**1.311 (1.107–1.707)**	**0.019**	**1.281 (1.105–1.321)**	**0.042**
CA	18 (4.8)	29 (7.7)	0.828 (0.441–1.555)	0.556	0.820 (0.427–1.575)	0.551
CT	8 (2.1)	8 (2.1)	0.510 (0.187–1.393)	0.182	0.602 (0.211–1.718)	0.343
Tobacco habit						
UP	14 (3.7)	97 (25.7)	1.449 (0.761–2.759)	0.257	1.580 (0.818–3.055)	0.173
MY	18 (4.8)	38 (10.1)	0.485 (0.195–1.205)	0.082	0.261 (0.103–1.365)	0.094
CA	5 (1.3)	42 (11.1)	1.680 (0.636–4.438)	0.291	1.693 (0.640–4.482)	0.289
CT	6 (1.6)	10 (2.7)	0.293 (0.102–0.840)	0.016	0.349 (0.119–1.025)	0.055

AFSI, age of first sexual intercourse; MSP, multiple sexual partners; STI, Sexually Transmitted Infections; OR, odds ratios; CI, confidence intervals. Chi^2^ test was used to compare between groups. A *p*-value < 0.5 was considered significant. ± ORs and *p*-values adjusted for age and clinical variables.

## Data Availability

Not applicable.

## Supplementary Materials

The following are available online at https://www.mdpi.com/article/10.3390/pathogens10121618/s1. Supplementary Table S1: Primers sequences.
