# Supplementary material for: Monitoring Sexually Transmitted Infections in Cervicovaginal Exfoliative Samples in Mexican Women
Source: Pathogens. 2021 Dec 13;10(12):1618. doi: 10.3390/pathogens10121618 (PMC8703509; doi:10.3390/pathogens10121618)
Supplement: Supplementary file 1 [file pathogens-10-01618-s001.zip › pathogens-1466694-supplementary.pdf]

Supplementary Table S1. Primers sequences.

Pathogen	**Primer Name**	**Sequence**
Mycoplasma spp.	M2_Myspp_F1	CACACCGTAAACGATAGATACT
	M2_Myspp_F2	CACGCCGTAAACGATGATCATT
	M2_Myspp_R1	AGTCTAGGTAAGGTTTTTCGTG
	M2_Myspp_R2	GAGTGGGTAAGGTTTTCCGTGT
Ureaplasma spp.	M1_Uspp_F1	TCGCTCTTTGTGGCGACGCTTT
	M1_Uspp_R1	ACTTACAAACCCGCCTGCGCTC
Candida spp.	M1_Caspp_F1	CTTGGCTGGCCGGTCCATCTTT
	M1_Caspp_R1	AGCCTTGCGACCATACTCCCCC
Neisseria gonhorroeae	M1_Nego_F1	GCCGCGATAAACCGTGGGAACA
	M1_Nego_R1	CGGCTGTTGTGCGTTTGATGCC
Trichomonas vaginalis	M1_Trva_F1	ACCGAAACCTAGCAGAGGGCCA
	M1_Trva_R1	GACGCCTTCGCCTCAGCTTCTC
Human Herpes Virus	M1_HSV_F1	GCCCTGGTCGACCTGCTGTTTT
	M1_HSV_R1	GTCTGCTCAGTTCGGCGGTGAG
